# Racial and immunologic disparities impact access to kidney rewaitlisting and retransplantation

**DOI:** 10.1016/j.ajt.2026.03.010

**Published:** 2026-03-16

**Authors:** Brendan P. Lovasik, Cristin Garrett, Rebecca Zhang, Mengyu Di, Raymond J. Lynch, Rachel E. Patzer, Andrew B. Adams

**Affiliations:** 1 Department of Surgery, Washington University School of Medicine, St. Louis, Missouri, USA; 2 Emory Transplant Center, Emory University, Atlanta, Georgia, USA; 3 Rollins School of Public Health, Emory University, Atlanta, Georgia, USA; 4 Reginstrief Institute, Indianapolis, Indiana, USA; 5 Health Systems Bureau, Health Resources and Services Administration, Rockville, Maryland, USA; 6 Department of Surgery, University of Minnesota, Minneapolis, Minnesota, USA

**Keywords:** retransplantation, racial disparities

## Abstract

The number of patients with a failed kidney transplant who are being considered for retransplantation is steadily increasing. However, disparities in access to repeat waitlisting and retransplant have not been well described. We examined a cohort of 102 891 patients with a failed kidney transplant in the United States Renal Data System (1995–2022) and assessed access to repeat waitlisting and repeat transplantation. Approximately half of the study population was relisted after graft failure, including similar proportions of White (49.6%), Black (46.8%), and Hispanic patients (49.9%). There were no differences in rewaitlisting of either Black (hazard ratio [HR], 1.00; 95% confidence interval [CI], 0.97–1.02) or Hispanic (HR, 1.01; 95% CI, 0.98–1.04) patients regardless of sociodemographics or clinical covariables. Interestingly, White patients had a higher rate of retransplantation (30.9%) than Black (20.8%; HR, 0.67; 95% CI, 0.65–0.69) or Hispanic (26.8%; HR, 0.78; 95% CI, 0.75–0.81) patients when sociodemographics or clinical covariables were controlled. When stratified by recipient panel reactive antibody, racial disparities were reduced; however, moderate and highly sensitized Black and Hispanic patients had reduced access to retransplant from the waiting list. Access to the waitlist alone does not account for racial/ethnic differences in retransplantation; policies and strategies addressing both sociodemographic and immunologic disparities should be promoted to improve access to kidney retransplant for minority patients.

## 1. Introduction

Renal transplantation is the preferred treatment for patients with end-stage renal disease (ESRD) due to its benefits in mortality, cost, and quality of life vs dialysis.^1–3^ Although short-term outcomes after renal transplantation are improving, long-term graft failure rates have remained fairly constant for several decades, and allograft loss and subsequent return to ESRD remains a significant issue in the kidney transplant community.^1,4,5^ However, many patients with a failed allograft have the opportunity to return to the kidney waiting list for the chance at another transplant, and retransplantation still offers a survival advantage compared with remaining on dialysis after a failed transplant.^6–10^

An increasing number of kidney transplants are being performed in retransplant recipients. Over 12 600 patients on the kidney waiting list are awaiting repeat kidney transplant, representing 12% of all patients on the waiting list; there has been a 50% increase in the number of retransplants performed from a decade prior. Despite recent changes in the kidney allocation system that prioritize sensitized individuals, retransplant patients with higher panel reactive antibody (PRA) levels have been associated with longer waiting times and have worse retransplant outcomes.^11–14^

Numerous studies have documented racial/ethnic and socioeconomic disparities in access to primary kidney transplant in the United States.^15–32^ Among those disparities, Black patients with ESRD are less likely to be waitlisted or to receive a transplant compared with White patients.^30,33,34^ However, less is known regarding whether these racial/ethnic disparities exist in access to repeat kidney transplant among all patients with a failed allograft.

Given the growing numbers of patients awaiting kidney retransplantation, it is critical to define the barriers that may exist to ensure equity in access. The aim of this study was to define if recipient sociodemographic and clinical disparities exist in access to kidney retransplantation, and if so, to determine the underlying etiologies of these disparities. We hypothesized that, similar to primary kidney transplants, race and socioeconomic factors are important determinants in access to repeat kidney transplantation for patients with ESRD.

## 2. Methods and analyses

### 2.1. Study population and data sources

All patient information was obtained from the United States Renal Data System (USRDS), a comprehensive national data registry of ESRD patients in the United States. Using the 2023 USRDS Standard Analysis Files (SAFs), we identified 102 891 patients who received their primary kidney transplant between January 1, 1995, and August 31, 2022, and subsequently had a graft failure before August 31, 2022. The graft failure date was defined as a return to dialysis after the initial transplant, receipt of another transplant, or the date provided by the SAF. Patients who received another kidney transplant either from an unknown donor source or with incomplete listing information were excluded.

Sociodemographic and clinical information, including patient age, sex, race, comorbidities, cause of ESRD, date of dialysis start, and dialysis modality at time of dialysis initiation, were obtained from the Centers for Medicare & Medicaid Services Form 2728 within the USRDS data. Additional transplant characteristics such as donor type, donor race, human leukocyte antigen (HLA) mismatch, and comorbidities were obtained from the United Network for Organ Sharing transplant files. The demographic data, including zip codes, provided in the USRDS file was then linked to the US Census to provide data on county-level poverty. This study was reviewed and approved by the Emory University Institutional Review Board (IRB00053157), and this manuscript was formulated in accordance with the Strengthening the Reporting of Observational Studies in Epidemiology (STROBE) statement for reporting of observational studies.

### 2.2. Study variables

The primary outcome was receipt of a repeat kidney transplant, either from a deceased or living donor, before August 31, 2022. Receipt of retransplant was defined by a second or third transplant date and was stratified by donor type in analyses, with time origin of the retransplant analysis from the date of rewaitlisting. The primary exposure variable was recipient race/ethnicity, defined as White non-Hispanic, White Hispanic, and Black; these 3 categories composed approximately 95% of the failed allograft cohort. Recipient race/ethnicity ‘other’ was excluded. Secondary outcomes of interest included the impact of other sociodemographic and clinical recipient factors that have been shown to correlate with barriers to primary kidney transplant. We also examined key barriers in access to retransplantation, including time from graft failure to waitlist and time from waitlist to receipt of a deceased donor retransplant, or time between graft failure and receipt of a living donor transplant.

Degree of allosensitization was identified by calculated PRA (cPRA) and stratified into 3 groups based on the PRA at time of relisting: PRA = 0, representing patients with no detectable antibodies; PRA = 1–80, representing low to moderately sensitized patients; and PRA >80, representing highly sensitized patients.

### 2.3. Data analysis

Multivariable Cox proportional hazard models were used for access-to-transplant steps to determine which characteristics are more or less associated with progression toward a retransplant. The characteristics examined were race/ethnicity (main exposure) and included cofounder adjustments for sex, age, body mass index, insurance type, blood type, time on dialysis before first transplant, time from first transplant to graft failure, time from graft failure to waitlist, percentage of poverty, PRA at time of second transplant, and temporary inactivity (Status 7) during time of waitlist. Comorbidities at the time of transplantation, as well as patient age, sex, and race, were obtained from the SAF 2022 transplant file, along with medical insurance, body mass index, and clinical characteristics that were documented at treatment start on Centers for Medicare & Medicaid Services Form 2728. Dialysis vintage was defined as the number of years from ESRD initiation to the first transplant. Neighborhood poverty information was obtained from the US Census.

We developed an a priori causal direct acyclic graph model based on known factors associated with access to transplantation, both initial and subsequent retransplantation, as described in the Supplementary Materials. Based on the causal direct acyclic graph, a Cox proportional hazards analysis was performed to investigate the influence of covariates including race/ethnicity on likelihood of rewaitlisting or repeat transplant. In overall access to transplant, patients were censored at death or study end. In the case of deceased donor transplant outcome, subjects were censored at the time of death or receipt of a living donor transplant if a patient did not receive one from a deceased donor. To elucidate the effect of pre-emptive retransplantation, we performed subgroup analyses among those who were rewaitlisted and retransplanted with a documented return-to-dialysis date against those with no return-to-dialysis date, signifying pre-emptive rewaitlisting.

## 3. Results

### 3.1. Characteristics of the study population

A total of 102 891 patients meeting inclusion criteria received a transplant with subsequent graft failure during the study period. Of these patients, 51.1% were White, 35.4% were Black, and 13.5% were Hispanic (Table 1). The most common causes of ESRD in White patients were glomerular nephritis (26.6%), diabetes (26.2%), and hypertension (20.1%). The mean age of the entire cohort at the time of first transplant was 43.3 years, with Hispanic patients being slightly younger (40.3 years), and the mean age at the time of first graft failure was 49.6 years.

### 3.2. Access to the waitlist for retransplant is not influenced by recipient race/ethnicity

To evaluate the likelihood of repeat kidney transplantation, we first examined key proximal barriers in access to retransplantation. Approximately half of the study population was relisted after graft failure, including similar proportions of White (49.6%), Black (46.8%), and Hispanic patients (49.3%) (Table 2). Patients with private health insurance were more likely to be represented in the waitlisted group than those with public or no insurance. Additionally, a higher percentage of patients with comorbidities were never rewaitlisted (Table 2).

Pre-emptive rewaitlisting (ie, waitlisting prior to return to dialysis in the setting of a failing allograft) accounted for 13 561 patients, or 27.1% of all patients who were rewaitlisted (Supplementary Table 1). Pre-emptively rewaitlisted patients were more likely to be White (60.4% vs 52.2%) and privately insured (44.6% vs 40.1%) than all rewaitlisted patients, as well as had a shorter interval from time of initial transplant to graft failure (1.87 years) and interval from graft failure to retransplant (2.54 years). Among pre-emptively rewaitlisted patients, White patients had a shorter interval from allograft failure to retransplant than either Black or Hispanic patients (2.23 vs 3.37 vs 2.74 years, *P* < .001).

### 3.3. Race/ethnicity influences the likelihood of retransplant independent of socioeconomic status

**Figure 1.**
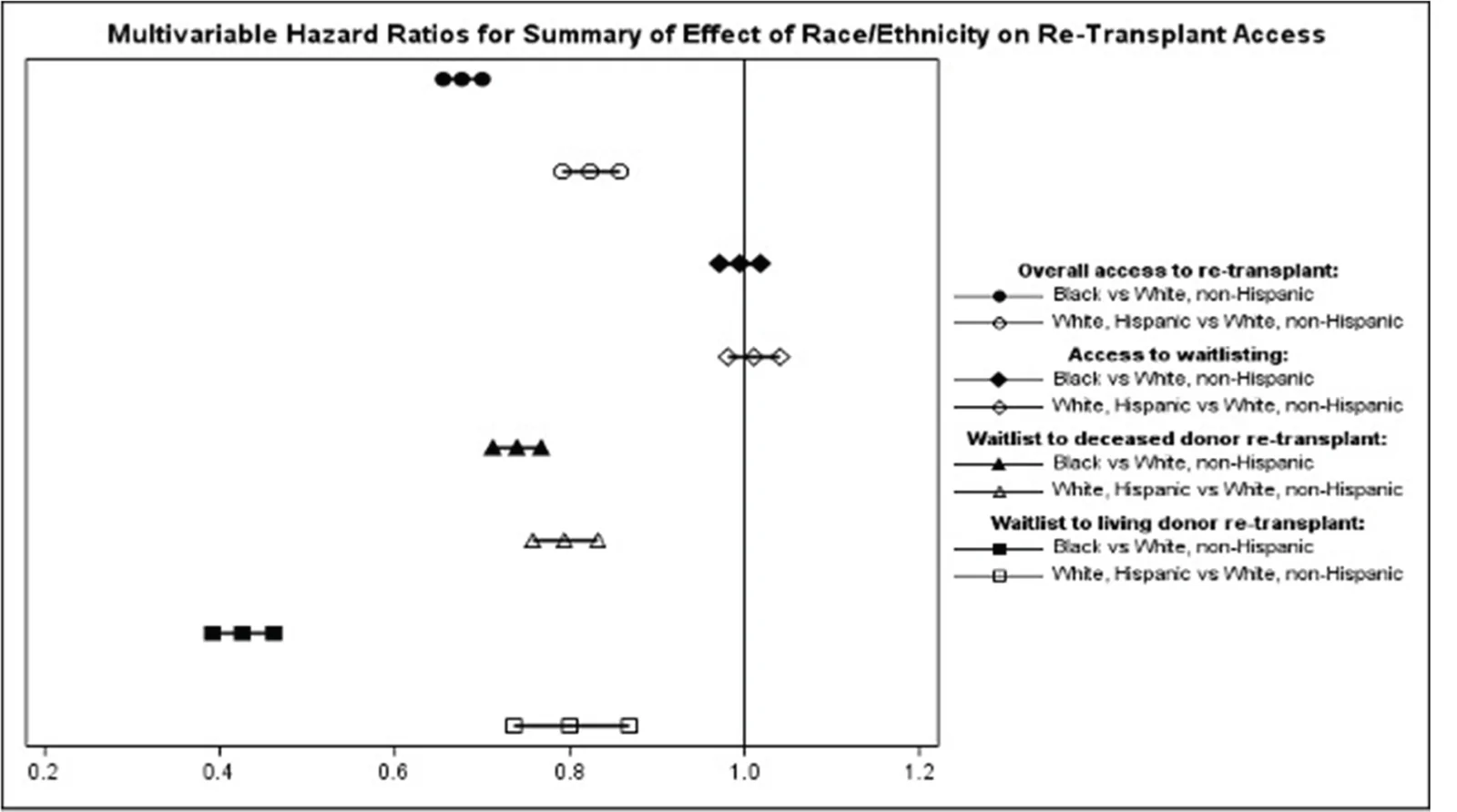
Forest plot of race/ethnicity effects on access to retransplant steps, including rewaitlisting or retransplant. On the x-axis, hazard ratio = 1.0 denotes equal likelihood of achieving retransplant step relative to White non-Hispanic patients; y-axis denotes stratification by each race effect.

**Figure 2.**
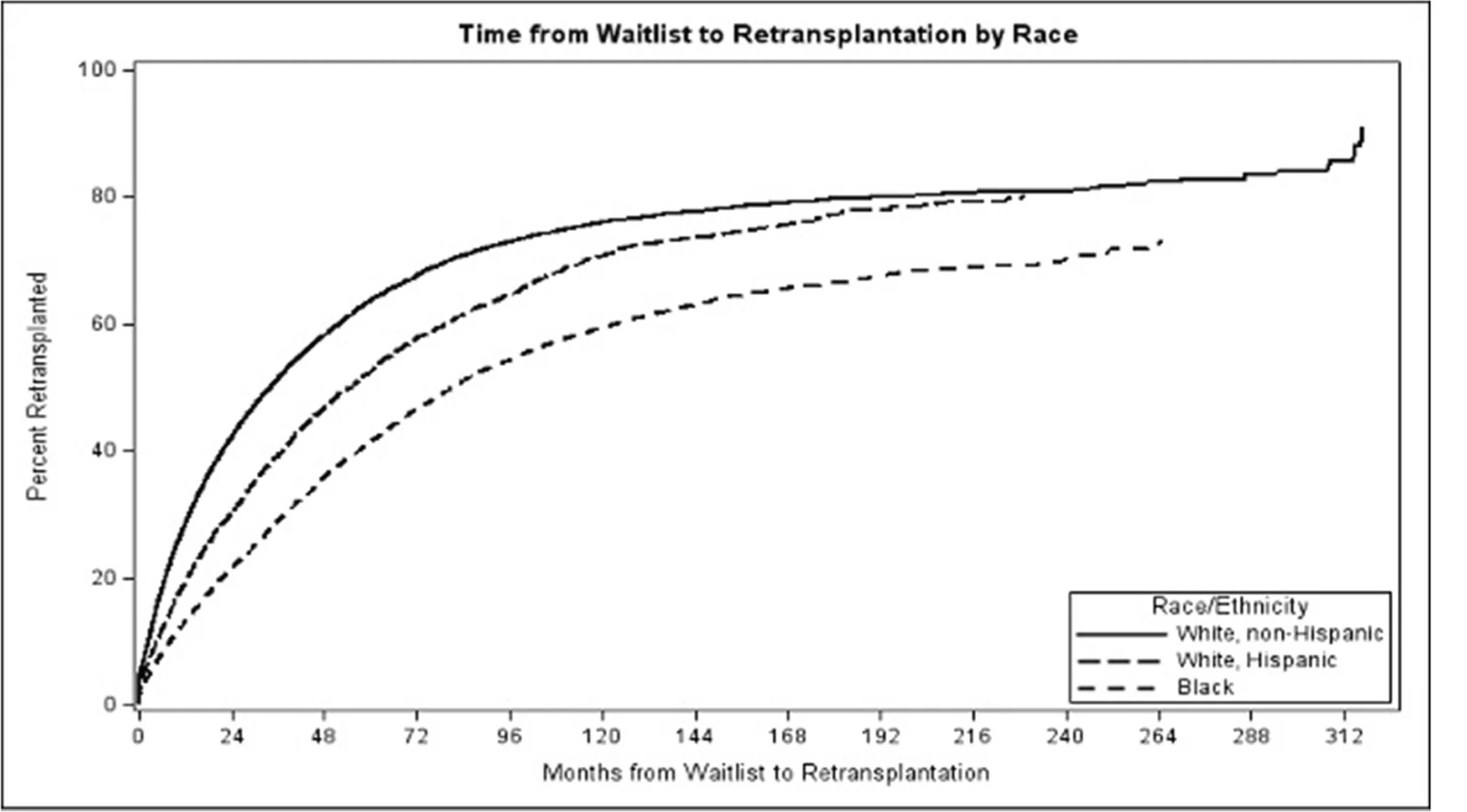
Percentage of rewaitlisted patients receiving deceased donor retransplant, stratified by time on waiting list and recipient race.

Overall, White patients were more likely to receive a retransplant and to receive retransplant earlier than either Black or Hispanic patients. White patients had a higher overall rate of retransplantation (30.9%) than Black (20.8%) or Hispanic (26.8%) patients. In adjusted models, Black patients were still less likely to receive a repeat transplant than their White counterparts (hazard ratio [HR], 0.68; 95% confidence interval [CI] 0.59–0.63) (Fig. 1, Table 3). Hispanic patients were also less likely to undergo retransplantation compared with White patients (HR, 0.82; 95% CI, 0.79–0.86). The median time from graft failure to retransplantation was 26.6 months (interquartile range [IQR], 10.7–52.9 months); however, this waiting time varied by race: among Black patients, the median time was 40.8 months (IQR, 18.7–69.5 months) vs 20.5 months (IQR, 8.3–42.0 months) among White patients (Fig. 2). Additionally, a higher percentage of patients with comorbidities were never retransplanted (Table 2).

We further defined access to retransplant by stratifying the transplanted patients by receipt of either a deceased or living donor organ. The percentage of Black patients receiving a living donor retransplant (14.2%) was significantly lower than either White (36.4%) or Hispanic patients (24.6%) (*P* < .001). Black patients were significantly less likely (HR, 0.42; 95% CI, 0.39–0.46) to receive a living donor kidney retransplant than White patients (Fig. 1, Table 3). This trend was observed for Hispanic patients as well, but to a lesser degree (HR, 0.80; 95% CI, 0.74–0.87). To assess the contribution of living donation to diminished overall access to retransplant, we next examined the influence of race on receipt of a deceased donor retransplant. Similar to overall access to retransplant, we found that Black and Hispanic patients were significantly less likely to receive a deceased donor retransplant than White patients, with HRs of 0.74 (95% CI, 0.71–0.77) and 0.79 (95% CI, 0.76–0.83), respectively. When the likelihood of rewaitlisting was evaluated, we found that recipient race was a less significant contributor. The impact of race on rewaitlisting was mitigated when socioeconomic status was controlled for by adjusting for insurance and poverty level and was eliminated altogether when all covariates were fully adjusted (Black patients: HR, 1.00; 95% CI, 0.97–1.02; Hispanic patients: HR, 1.01; 95% CI, 0.98–1.04). When we subclassified these models by patients who resumed dialysis prior to retransplant or those who received pre-emptive retransplant, we found that the same disparities existed (Table 3; Supplementary Fig. S2A, B). Results were similar, with Black and Hispanic patients showing overall lower rates of retransplantation than White patients. Interestingly, pre-emptive waitlisting appeared to have a beneficial effect on Black patients’ access to deceased donor retransplantation (HR 0.83 vs 0.67), although it did not reach statistical significance; however, this trend was not seen in Black patients’ access to living donor retransplantation (HR 0.40 vs 0.43). Hispanic patients showed no difference in access to either deceased (HR 0.78 vs 0.74) or living donor (0.79 vs 0.82) retransplantation.

### 3.4. Race was a more significant contributor to access to retransplant than medical comorbidities

Although race/ethnicity had little influence on whether a patient was rewaitlisted, race/ethnicity demonstrated a significant impact on the likelihood of retransplant in a multivariable Cox proportional hazards analysis. When controlling for all covariates, Black patients had a lower likelihood of retransplant (HR, 0.67; 95% CI, 0.65–0.69) than White patients (Table 4). Hispanic patients were also less likely to receive a retransplant than White patients (HR, 0.78; 95% CI, 0.75–0.81). When these models were subclassified by patients who resumed dialysis prior to retransplant or those who received pre-emptive retransplant, similar findings were largely observed (Supplemental Table 2).

One possible hypothesis for lower rates of relisting was that some patients were ‘too sick’ to relist. Comorbidities including congestive heart failure, chronic obstructive pulmonary disease, cardiovascular disease, and diabetes mellitus appeared to influence the decision to rewaitlist a candidate (Table 2) but played less of role in the decision to retransplant once the patient was on the waitlist. Race/ethnicity had a significantly greater negative impact on the likelihood of overall access to retransplant, particularly for Black patients, than any medical comorbidities, with Black patients being nearly 2-fold less likely to receive a retransplant than White patients with diabetes mellitus, peripheral vascular disease, congestive heart failure, or chronic obstructive pulmonary disease. Dialysis vintage and neighborhood poverty level were both associated with reduced access to waitlisting and retransplantation, whereas group insurance status and longer primary graft survival were associated with higher rates of retransplantation (Table 4).

Unsurprisingly, patients with blood types B and O were less likely to receive a retransplant, whereas patients with an AB blood type were more likely to receive a retransplant. Given the higher percentage of blood type B in the Black population, we performed a sensitivity analysis to evaluate the impact of race and blood type distinctly but found no significant interaction between race and blood type (data not shown).

### 3.5 Race and PRA combine to limit access to retransplant

We hypothesized that previously transplanted recipients with a failed allograft are also much more likely to develop anti-HLA antibodies and have a higher PRA level, which may differentially impact access to retransplant.^35^ We identified a notable interaction between race and PRA (Table 5) in which Black and Hispanic patients were less likely to undergo repeat kidney transplant at any PRA level compared with White patients; this disparity was exacerbated in the low/moderately (PRA 1–80) and highly (PRA >80) sensitized patients. In fact, highly sensitized (PRA >80) Black patients were almost half as likely to receive a deceased donor transplant (HR, 0.65; *P <* .001) relative to highly sensitized White patients despite the same PRA category.

### 3.6. Donor/recipient compatibility by race and HLA type likely contribute to influence access to retransplant

To further examine the race-based differences between White and Black transplant recipients shown above, we evaluated the distribution of recipient and donor race/ethnicity from their initial and repeat transplants (Table 6). White donors made up the majority of donors for both primary and repeat transplant; as such, White patients were more likely to receive a from a donor of their same race (84.4%), in contrast to Black patients receiving an organ from a donor of their same race (34.4%). This association was also true for repeat kidney transplantation (83.9 vs 30.8%).

Finally, we examined the association between the level of HLA mismatch and race for both the primary and repeat transplants. A notably higher percentage of White patients received a 0-antigen mismatched kidney compared with Black patients (8.3% vs 3.0%) for their initial transplant (Table 7). These discrepancies were also seen in patients undergoing repeat transplantation, in whom the percentage of White patients receiving a 0-mismatched kidney was nearly 3-fold higher than that of Black patients (15.6% vs 5.6%). Black patients were more likely to receive a highly mismatched (5–6 mismatch) kidney at the time of primary (49.3 vs 35.6%) and repeat transplantation (41.7 vs 27.7%) than White patients.

### 3.7. Racial disparities exist in access to rewaitlisting and retransplant among patients with ≥2 failed allografts

Among all patients with failed allografts, 73 847 (72%) had 1 previous kidney transplant, 27 002 (26%) had 2 previous transplants, and 2042 (2%) had ≥3 previous kidney transplants. Subgroup analysis of patients with ≥2 failed allografts (ie, consideration for a third kidney retransplant) is shown in Table 8. Of note, Black (0.94; 95% CI, 0.91–0.97; *P <* .001) and Hispanic patients (0.94; 95% CI, 0.90–0.97; *P* < .001) were less likely to be rewaitlisted than White patients; this disparity was not seen among patients with only a single failed allograft. Black and Hispanic patients with ≥2 failed allografts were also less likely than White patients to undergo retransplantation, and this disparity was greater than for a single failed graft.

## 4. Discussion

In this national study of kidney transplant patients requiring retransplant, Black and Hispanic patients were less likely to undergo rewaitlisting and retransplantation than White patients. These racial/ethnic disparities in access to waitlisting were nearly eliminated when controlling for comorbidities and socioeconomic factors; however, there were still significant disparities in access to both deceased and living donor retransplantation even after patients were waitlisted. Interestingly, among patients with ≥2 failed allografts, there were disparities in access to both rewaitlisting and retransplantation among both Black and Hispanic patients. These disparities persisted despite controlling for both return to dialysis or pre-emptive retransplantation. These data suggest that although race plays a critical role in access to retransplantation, it does not demonstrate a dominant influence on rewaitlisting to the exclusion of other factors including socioeconomic status. Perhaps most interestingly, there was a notable association with PRA sensitization and race, such that highly sensitized Black patients were almost half as likely to receive a deceased donor transplant relative to highly sensitized White patients despite the same PRA category, and Black and Hispanic patients were more likely to receive an organ with higher HLA mismatch for their repeat transplant. The data presented here suggest that immunologic factors may play a role in racial disparities in access to retransplantation and pose a significant barrier to minority patients receiving a repeat transplant even once they have been rewaitlisted.

There are several possible explanations for these findings. First, most donor organs are from White patients who may be better matched and thus have higher priority for White recipients. Given the differences in frequency of HLA alleles between members of each race, this propensity for Black recipients to receive a kidney from a White donor may contribute to sensitization of Black recipients against White donor-predominant HLA types that occurs with the first failed kidney transplant. This higher degree of sensitization could then lead to higher rates of rejection, worse graft and patient outcomes, and the need for retransplant with a still more difficult match.^36–41^ Hispanic patients are likely less impacted than Black patients because the HLA antigen frequencies for Hispanic alleles/haplotypes is closer to those of White patients, which can differ significantly from those of Black patients.^42^ A recent study examining of referrals and evaluations among potential deceased donors demonstrates that patterns of donor evaluation and consent for organ donation were lower among potential Black organ donors than potential White organ donors; taken together with the results of this study, these data suggest that disparities in recipient matching are also influenced by availability of donors with similar HLA profiles.^43,44^

In our subgroup analysis of blood groups among Black patients, we found no association blood group B and retransplant rate. A recent study by Gragert et al^45^ quantified the disparity in cPRA among different blood groups as an ‘ABO-adjusted cPRA’ by combining HLA-compatible donors among the ABO-compatible donor pool; this calculation was found to increase the adjusted cPRA among all patients with blood group B, which may increase their priority for PRA-based allocation and improve access for retransplantation. The data in this study also suggest that although sensitized patients generally have lower access to retransplantation, this is not true for those underwent pre-emptive retransplantation. Several recent advances may be promising in addressing the sensitization problems in patients awaiting retransplant, including immunologic desensitization protocols for high PRA recipients, broader sharing of deceased donor organs for sensitized patients, and living donor paired exchange programs to improve donor-recipient crossmatches.^36^

African Americans are also less likely to receive transplants from living donors for both first and second transplants. In our study population, Black patients were less likely to receive a living donor kidney for their retransplant (14%) than White patients (36%). This represents another disparity in quality of care among minority patients, as living donor retransplants are associated with higher graft survival than deceased donor retransplants at 1, 3, and 5 years.^36^ It is also notable that among patients with ≥2 failed allografts, both Black and Hispanic patients were less likely to be rewaitlisted or undergo retransplantation; this disparity in waitlisting was not seen among patients with only a single failed allograft. The subgroup of multitransplant patients, though relatively small, is an interesting cohort as they are well integrated into the transplant infrastructure, though they may be more medically complex and highly sensitized from prior transplant episodes; this population deserves further study.

These data are particularly important and timely as the number of patients who will need a repeat kidney transplant will increase over the coming years. Heaphy et al^46^ demonstrated impaired access to the waiting list among minority retransplant patients, and Schold et al^47^ recently reported racial disparities in access to the retransplant waiting list for pre-emptive retransplantation; however, the study only included outcomes of pre-emptive relisting and did not include rates of retransplantation from the waiting list. In our data, over a quarter (27%) of patients in the sample were pre-emptively rewaitlisted (ie, waitlisted prior to return to dialysis); this high rate of pre-emptive rewaitlisting is interesting given the known access-to-transplant disparities and improved outcomes associated with pre-emptive transplantation that have been observed in primary transplantation. Sandal et al^48^ recently reported disparities in relisting and retransplant among Black and Hispanic patients with a failed allograft; however, the study did not include the immunologic parameters that are contribute significantly to receipt of repeat kidney transplantation.

The limitations of this study should be noted. First, the retrospective nature of this review is subject to expected limitations, and causality and mechanisms cannot be established; further, there may be other cofounding factors not codified that may explain certain associations. Second, the study period includes changes to the US kidney allocation system in December 2014, which includes prior organ transplant in its calculation of expected posttransplant survival, which may impair access to retransplant^10,49^; conversely, this program improves access for highly sensitized patients, which may indirectly benefit retransplant patients.

Despite these limitations, there are a number of strengths to our analyses. This study was the first to our knowledge to examine both the sociodemographic and immunologic disparities in repeat kidney transplantation. Further, use of a nationally standardized national data set over a 27-year period contributes to validity and generalizability of these findings when developing interventions to mitigate these disparities. Future directions of study include analysis of the impact of donor-recipient race and HLA mismatches and how these may contribute to allosensitization.

In this study, patient race was not a factor in access to the waitlist among patients awaiting repeat transplantation, but it does have a significant impact on the likelihood of receiving a repeat transplant while on the waitlist. Further, immunologic disparities play a significant role in access to repeat kidney transplantation. Further investigation of the biologic origins of these disparities given the differences in HLA type between donor and recipient races are needed to develop appropriate kidney allocation strategies and improve patient care for patients requiring repeat kidney transplant.

## Supplementary Material

1

2

3

4

## Figures and Tables

**Table 1:** Patient characteristics of patients with a failed kidney allograft eligible for retransplant in the United States, 1995–2022.

Characteristic	Study population N = 102 891	White, non-Hispanic N = 52 598	Black N = 36 377	White, Hispanic N = 13 916	*P*

Cause of ESRD					
Diabetes	26 927 (26.17)	14 287 (27.16)	8561 (23.53)	4079 (29.31)	<.001**
Hypertension	20 633 (20.05)	5994 (11.4)	12 334 (33.91)	2305 (16.56)	<.001**
Glomerular nephritis	28 368 (27.57)	14 614 (27.78)	9723 (26.73)	4031 (28.97)	<.001**
Cystic kidney	6671 (6.48)	5128 (9.75)	967 (2.66)	576 (4.14)	<.001**
Other urologic	4127 (4.01)	2947 (5.6)	649 (1.78)	531 (3.82)	<.001**
Other cause	11 166 (10.85)	7170 (13.63)	2602 (7.15)	1394 (10.02)	<.001**
Unknown cause	4739 (4.61)	2311 (4.39)	456 (4)	972 (6.98)	<.001**
Missing cause	260 (0.25)	147 (0.28)	85 (0.23)	28 (0.2)	.175
Insurance					
Group health insurance	33 630 (32.69)	20 299 (38.59)	10 172 (27.96)	3159 (22.7)	<.001**
Medicaid	24 419 (23.73)	9022 (17.15)	10 177 (27.98)	5220 (37.51)	<.001**
Medicare or other insurance	36 192 (35.18)	20 227 (38.46)	11 847 (32.57)	4118 (29.59)	<.001**
No health insurance	3096 (3.01)	1038 (1.97)	1725 (4.74)	333 (2.39)	<.001**
Unknown	5554 (5.40)	2012 (3.83)	2456 (6.75)	1086 (7.8)	<.001**
Male	61 473 (59.75)	31 605 (60.09)	21 606 (59.39)	8262 (59.37)	.073
CAD	6902 (7.34)	4391 (9.1)	1678 (5.1)	833 (6.44)	<.001**
CHF	14 903 (15.84)	7891 (16.35)	5259 (15.99)	1753 (13.56)	<.001**
PVD	6407 (6.81)	3825 (7.92)	1725 (5.25)	857 (6.63)	<.001**
COPD	2759 (2.93)	1843 (3.82)	739 (2.25)	177 (1.37)	<.001**
Diabetes	35 368 (35.65)	18 004 (35.23)	12 237 (35.41)	5127 (37.84)	<.001**
Age, y, mean (SD)	43.28 (16.02)	43.76 (16.5)	43.74 (14.8)	40.28 (16.87)	<.001**
Duration of primary transplanted graft, y, mean (SD)	28.37 (52.70)	28.07 (53.29)	29.16 (60.2)	27.49 (19.73)	<.001**
Interval from graft failure to retransplantation, y, mean (SD)	6.29 (5.28)	6.74 (5.58)	5.52 (4.72)	6.64 (5.27)	<.001**

Data are n (%) unless otherwise specified.

CAD, coronary artery disease; CHF, congestive heart failure; COPD, chronic obstructive pulmonary disease; ESRD, end-stage renal disease; PVD, peripheral vascular disease; SD, standard deviation.

** *P* < .05.

**Table 2:** Sociodemographic characteristics of patients with a failed kidney allograft eligible for retransplant stratified by retransplant steps, including rewaitlisting or retransplant.

Characteristic	Study population N = 102 891	Neither waitlisted nor retransplanted N = 51 557	Waitlisted N = 49 998	Retransplanted N = 27 571			*P* ^a^
				All retransplants	Deceased donor retransplant	Living donor retransplant	

Race/ethnicity							<.001**
White, non-Hispanic	52 598	25 463 (48.41)	26 105 (49.63)	16 265 (30.92)	10 340 (63.57)	5925 (36.43)	
Black	36 377	19 143 (52.62)	17 028 (46.81)	7566 (20.8)	6491 (85.79)	1075 (14.21)	
White, Hispanic	13 916	6951 (49.95)	6865 (49.33)	3740 (26.88)	2817 (75.32)	923 (24.68)	
Cause of primary ESRD							<.001**
Diabetes	26 927	17 788 (66.06)	8805 (32.7)	3705 (13.76)	2503 (67.56)	1202 (32.44)	
Hypertension	20 633	11 496 (55.72)	8993 (43.59)	4210 (20.4)	3437 (81.64)	773 (18.36)	
Glomerular nephritis	28 368	11 091 (39.1)	16 867 (59.46)	10 248 (36.13)	7178 (70.04)	3070 (29.96)	
Cystic kidney	6671	2869 (43.01)	3725 (55.84)	2142 (32.11)	1525 (71.2)	617 (28.8)	
Other urologic	4127	1433 (34.72)	2602 (63.05)	1773 (42.96)	1178 (66.44)	595 (33.56)	
Other cause	11 166	4815 (43.12)	6162 (55.19)	3804 (34.07)	2605 (68.48)	1199 (31.52)	
Unknown cause	4739	1968 (41.53)	2709 (57.16)	1604 (33.85)	1186 (73.94)	418 (26.06)	
Missing cause	260	97 (37.31)	135 (51.92)	85 (32.69)	36 (42.35)	49 (57.65)	
Insurance							<.001**
Group health insurance	33 630	12 896 (38.35)	20 067 (59.67)	12 056 (35.85)	7759 (64.36)	4297 (35.64)	
Medicaid	24 419	12 868 (52.7)	11 346 (46.46)	5983 (24.5)	4832 (80.76)	1151 (19.24)	
Medicare or other insurance	36 192	21 222 (58.64)	14 628 (40.42)	7662 (21.17)	5575 (72.76)	2087 (27.24)	
No health insurance	3096	1717 (55.46)	1350 (43.6)	571 (18.44)	468 (81.96)	103 (18.04)	
Unknown	5554	2854 (51.39)	2607 (46.94)	1299 (23.39)	1014 (78.06)	285 (21.94)	
Male	61 473	31 180 (50.72)	29 564 (48.09)	16 145 (26.26)	11 650 (72.16)	4495 (27.84)	<.001**
Female	41 418	20 377 (49.2)	20 434 (49.34)	11 426 (27.59)	7998 (70)	3428 (30)	.021**
CAD	6902	4694 (68.01)	2151 (31.16)	1004 (14.55)	746 (74.3)	258 (25.7)	<.001**
CHF	14 903	10 388 (69.7)	4404 (29.55)	2019 (13.55)	1534 (75.98)	485 (24.02)	.046**
PVD	6407	4578 (71.45)	1773 (27.67)	777 (12.13)	577 (74.26)	200 (25.74)	<.001**
COPD	2759	2049 (74.27)	694 (25.15)	327 (11.85)	267 (81.65)	60 (18.35)	.859
Diabetes	35 368	22 083 (62.44)	12 881 (36.42)	6017 (17.01)	4279 (71.12)	1738 (28.88)	<.001**
Duration of primary transplanted graft, y, mean (SD)	43.28 (16.02)	48.61 (14.88)	38.02 (15.31)	34.87 (15.27)	35.65 (15.41)	32.91 (14.71)	.033**
BMI, kg/m^2^, mean (SD)	28.37 (52.70)	28.97 (67.06)	27.78 (32.83)	27.32 (20.77)	27.49 (17.59)	26.9 (27.06)	<.001**

Data are shown as n (%) unless otherwise specified.

BMI, body mass index; CAD, coronary artery disease; CHF, congestive heart failure; COPD, chronic obstructive pulmonary disease; ESRD, end-stage renal disease; PVD, peripheral vascular disease; SD, standard deviation.

a *P* values are testing the proportion of deceased donor/living donor transplant.

** *P* < .05.

**Table 3:** Multivariable hazard ratios for effect of race/ethnicity on access to retransplant steps, including rewaitlisting or retransplant.

Comparitor category	Effect of race/ethnicity, hazard ratio (95% CI)		
	Crude model	Covariates adjusted except insurance and poverty level	Covariates fully adjusted

All patients with failed primary kidney transplant			
Overall access to DD or LD retransplant			
Black vs White, non-Hispanic	0.49 (0.48, 0.50)	0.61 (0.59, 0.63)	0.68 (0.66, 0.70)
White, Hispanic vs White, non-Hispanic	0.69 (0.67, 0.72)	0.72 (0.69, 0.75)	0.82 (0.79, 0.86)
Access to waitlisting			
Black vs White, non-Hispanic	0.81 (0.80, 0.83)	0.91 (0.89, 0.93)	1.00 (0.97, 1.02)
White, Hispanic vs White, non-Hispanic	0.89 (0.86, 0.91)	0.90 (0.88, 0.93)	1.01 (0.98, 1.04)
Waitlist to retransplant			
Black vs White, non-Hispanic	0.53 (0.51, 0.55)	0.60 (0.58, 0.63)	0.63 (0.61, 0.66)
White, Hispanic vs White, non-Hispanic	0.78 (0.74, 0.81)	0.80 (0.76, 0.84)	0.85 (0.81, 0.89)
Waitlist to DD retransplant			
Black vs White, non-Hispanic	0.70 (0.68, 0.72)	0.74 (0.71, 0.76)	0.74 (0.71, 0.77)
White, Hispanic vs White, non-Hispanic	0.86 (0.83, 0.90)	0.79 (0.75, 0.82)	0.79 (0.76, 0.83)
Waitlist to LD retransplant			
Black vs White, non-Hispanic	0.22 (0.21, 0.24)	0.38 (0.35, 0.41)	0.42 (0.39, 0.46)
White, Hispanic vs. White, non-Hispanic	0.57 (0.53, 0.62)	0.69 (0.64, 0.74)	0.80 (0.74, 0.87)
Failed primary kidney transplant returned to dialysis			
Dialysis restart to waitlist			
Black vs White, non-Hispanic	0.75 (0.73, 0.77)	0.76 (0.74, 0.78)	0.79 (0.77, 0.81)
White, Hispanic vs White, non-Hispanic	0.78 (0.75, 0.80)	0.82 (0.79, 0.85)	0.86 (0.83, 0.89)
Dialysis restart to retransplant			
Black vs White, non-Hispanic	0.50 (0.48, 0.51)	0.57 (0.55, 0.59)	0.60 (0.58, 0.63)
White, Hispanic vs White, non-Hispanic	0.72 (0.69, 0.75)	0.76 (0.72, 0.79)	0.82 (0.78, 0.86)
Dialysis restart to DD retransplant			
Black vs White, non-Hispanic	0.58 (0.56, 0.60)	0.64 (0.61, 0.66)	0.66 (0.64, 0.69)
White, Hispanic vs White, non-Hispanic	0.78 (0.74, 0.82)	0.80 (0.76, 0.84)	0.85 (0.80, 0.89)
Dialysis restart to LD retransplant			
Black vs White, non-Hispanic	0.22 (0.20, 0.24)	0.32 (0.29, 0.36)	0.37 (0.33, 0.41)
White, Hispanic vs White, non-Hispanic	0.56 (0.51, 0.62)	0.66 (0.60, 0.73)	0.78 (0.70, 0.87)
Waitlist to retransplant			
Black vs White, non-Hispanic	0.53 (0.51, 0.55)	0.60 (0.58, 0.63)	0.63 (0.61, 0.66)
White, Hispanic vs White, non-Hispanic	0.78 (0.74, 0.81)	0.80 (0.76, 0.84)	0.85 (0.81, 0.89)
Waitlist to DD retransplant			
Black vs White, non-Hispanic	0.62 (0.60, 0.65)	0.67 (0.64, 0.70)	0.67 (0.65, 0.70)
White, Hispanic vs White, non-Hispanic	0.84 (0.80, 0.88)	0.76 (0.72, 0.80)	0.78 (0.74, 0.82)
Waitlist to LD retransplant			
Black vs White, non-Hispanic	0.22 (0.20, 0.25)	0.39 (0.35, 0.44)	0.43 (0.39, 0.48)
White, Hispanic vs White, non-Hispanic	0.59 (0.53, 0.65)	0.69 (0.62, 0.77)	0.79 (0.71, 0.88)
Failed primary kidney transplant with pre-emptive retransplant			
Waitlist to retransplant			
Black vs White, non-Hispanic	0.52 (0.50, 0.55)	0.63 (0.59, 0.66)	0.66 (0.62, 0.70)
White, Hispanic vs White, non-Hispanic	0.69 (0.64, 0.74)	0.72 (0.66, 0.77)	0.78 (0.72, 0.84)
Waitlist to DD retransplant			
Black vs White, non-Hispanic	0.71 (0.67, 0.76)	0.83 (0.77, 0.88)	0.83 (0.77, 0.89)
White, Hispanic vs White, non-Hispanic	0.74 (0.67, 0.81)	0.73 (0.66, 0.81)	0.74 (0.67, 0.82)
Waitlist to LD retransplant			
Black vs White, non-Hispanic	0.24 (0.22, 0.27)	0.36 (0.32, 0.41)	0.40 (0.35, 0.45)
White, Hispanic vs White, non-Hispanic	0.63 (0.56, 0.71)	0.70 (0.62, 0.79)	0.82 (0.72, 0.93)

CI, confidence interval; DD, deceased donor; LD, living donor.

**Table 4:** Multivariable Cox models for impact of various recipient sociodemographic and clinical factors on access to retransplant steps, including rewaitlisting or retransplant.

Covariate	Access to waitlisting	Access from waitlist to DD or LD retransplant	Access from waitlist to DD retransplant	Access from waitlist to LD retransplant	Overall access to DD or LD retransplant	Overall access to DD retransplant	Overall access to LD retransplant

Race							
White non-Hispanic	REF	REF	REF	REF	REF	REF	REF
Black	1.00 (0.97–1.02)	0.67 (0.65–0.69)***	0.74 (0.71–0.77)***	0.42 (0.39–0.46)***	0.68 (0.66–0.70)***	0.79 (0.76–0.82)***	0.41 (0.38–0.44)***
White Hispanic	1.01 (0.98–1.04)	0.78 (0.75–0.81)***	0.79 (0.76–0.83)***	0.80 (0.74–0.87)***	0.82 (0.79–0.86)***	0.89 (0.85–0.93)***	0.74 (0.69–0.80)***
Sex							
Male	REF	REF	REF	REF	REF	REF	REF
Female	1.00 (0.98–1.02)	1.01 (0.98–1.03)	0.98 (0.95–1.01)	1.08 (1.03–1.14)**	1.00 (0.98–1.03)	0.97 (0.95–1.00)	1.08 (1.03–1.13)**
CAD	0.93 (0.89–0.97)**	0.98 (0.91–1.05)	1.05 (0.97–1.14)	0.81 (0.70–0.94)**	0.93 (0.87–0.99)*	1.00 (0.93–1.09)	0.78 (0.68–0.89)***
CHF	0.74 (0.71–0.76)***	0.98 (0.94–1.03)	0.99 (0.94–1.05)	0.96 (0.86–1.07)	0.79 (0.76–0.83)***	0.81 (0.76–0.85)***	0.76 (0.69–0.84)***
COPD	0.69 (0.64–0.74)***	1.06 (0.94–1.19)	1.20 (1.06–1.36)**	0.63 (0.47–0.85)**	0.75 (0.67–0.84)***	0.87 (0.76–0.98)*	0.48 (0.37–0.63)***
Diabetes	0.78 (0.76–0.80)***	0.93 (0.90–0.96)***	0.98 (0.95–1.02)	0.82 (0.77–0.87)***	0.81 (0.79–0.84)***	0.83 (0.80–0.86)***	0.79 (0.75–0.84)***
PVD	0.77 (0.73–0.81)***	0.93 (0.86–1.00)	0.97 (0.89–1.06)	0.79 (0.66–0.93)**	0.76 (0.71–0.82)***	0.78 (0.72–0.85)***	0.71 (0.61–0.82)***
Age	0.99 (0.98–0.99)***	0.99 (0.99–0.99)***	0.99 (0.99–0.99)***	0.98 (0.98–0.98)***	0.98 (0.98–0.98)***	0.98 (0.98–0.98)***	0.97 (0.97–0.97)***
BMI	1.00 (1.00–1.00)	1.00 (1.00–1.00)	1.00 (1.00–1.00)	1.00 (1.00–1.00)	1.00 (1.00–1.00)*	1.00 (1.00–1.00)	1.00 (1.00–1.00)
Years ESRD initiation to first transplant	0.93 (0.93–0.94)***	0.96 (0.96–0.97)***	0.99 (0.99–1.00)	0.80 (0.78–0.82)***	0.89 (0.88–0.90)***	0.93 (0.92–0.94)***	0.72 (0.70–0.73)***
Years first transplant to graft failure	1.00 (1.00–1.00)**	1.01 (1.01–1.01)***	1.00 (0.99–1.00)*	1.03 (1.03–1.04)***	1.00 (1.00–1.01)***	0.99 (0.99–1.00)***	1.02 (1.02–1.03)***
Percentage below poverty	0.99 (0.99–0.99)***	1.00 (0.99–1.00)***	1.00 (1.00–1.00)	0.99 (0.98–0.99)***	0.99 (0.99–0.99)***	0.99 (0.99–0.99)***	0.98 (0.97–0.98)***
Insurance							
Group health insurance	1.38 (1.35–1.41)***	1.08 (1.05–1.12)***	1.00 (0.96–1.03)	1.28 (1.21–1.36)***	1.29 (1.25–1.33)***	1.16 (1.12–1.21)***	1.57 (1.48–1.66)***
Medicaid	0.89 (0.87–0.91)***	0.91 (0.88–0.95)***	0.98 (0.94–1.02)	0.71 (0.65–0.77)***	0.79 (0.76–0.82)***	0.84 (0.81–0.88)***	0.64 (0.59–0.69)***
Medicare or other insurance	REF	REF	REF	REF	REF	REF	REF
No insurance	1.05 (0.92–1.19)	0.93 (0.76–1.15)	0.99 (0.80–1.24)	0.62 (0.33–1.15)	0.80 (0.65–0.99)*	0.84 (0.67–1.05)	0.56 (0.31–0.98)*
Unknown insurance	0.81 (0.77–0.85)***	0.94 (0.88–1.01)	0.98 (0.91–1.06)	0.76 (0.63–0.91)**	0.68 (0.64–0.73)***	0.70 (0.64–0.75)***	0.59 (0.50–0.69)***
Recipient blood group							
A	REF	REF	REF	REF	REF	REF	REF
B	1.07 (1.04–1.10)***	0.73 (0.70–0.76)***	0.66 (0.62–0.69)***	0.99 (0.91–1.07)	0.78 (0.75–0.82)***	0.71 (0.68–0.75)***	1.00 (0.92–1.08)
O	1.03 (1.01–1.05)**	0.83 (0.81–0.86)***	0.82 (0.80–0.85)***	0.86 (0.82–0.91)***	0.88 (0.85–0.90)***	0.87 (0.85–0.90)***	0.89 (0.85–0.94)***
AB	1.01 (0.97–1.06)	1.14 (1.07–1.22)***	1.19 (1.11–1.28)***	1.00 (0.88–1.14)	1.11 (1.04–1.17)**	1.17 (1.09–1.25)***	0.96 (0.85–1.08)
Panel reactive antibody					
PRA = 0	—	REF	REF	REF	—	—	—
PRA (0, 80)	—	0.85 (0.82–0.88)***	1.16 (1.11–1.21)***	0.47 (0.45–0.50)***	—	—	—
PRA >80%	—	0.61 (0.58–0.64)***	0.83 (0.78–0.87)***	0.30 (0.26–0.33)***	—	—	—
Status 7	—	0.17 (0.16–0.18)***	0.10 (0.09–0.10)***	0.53 (0.50–0.56)***	—	—	—
Years from graft failure to waitlisting	—	1.03 (1.02–1.04)***	1.07 (1.06–1.07)***	0.85 (0.83–0.87)***	—	—	—

Data are shown as hazard ratio (95% confidence interval).

BMI, body mass index; CAD, coronary artery disease; CHF, congestive heart failure; COPD, chronic obstructive pulmonary disease; DD, deceased donor; ESRD, end-stage renal disease; LD, living donor; PRA, panel reactive antibody; PVD, peripheral vascular disease; REF, reference.

* *P* < .05.

** *P* < .01.

*** *P* < .001.

**Table 5:** Multivariable Cox models for impact of various recipient sociodemographic and clinical factors on access to retransplant steps, including rewaitlisting or retransplant.

Covariate	Access from waitlist to DD or LD transplant	Access from waitlist to DD transplant	Access from waitlist to LD transplant

PRA = 0			
White/non-Hispanic	REF	REF	REF
Black	0.74 (0.69–0.79)***	1.00 (0.93–1.09)	0.52 (0.46–0.59)***
White/Hispanic	0.73 (0.67–0.79)***	0.74 (0.67–0.83)***	0.88 (0.77–0.99)**
PRA (0, 80)			
White/non-Hispanic	REF	REF	REF
Black	0.65 (0.62–0.67)***	0.71 (0.68–0.74)***	0.35 (0.32–0.40)***
White/Hispanic	0.78 (0.74–0.82)***	0.80 (0.76–0.85)***	0.71 (0.64–0.80)***
PRA >80			
White/non-Hispanic	REF	REF	REF
Black	0.67 (0.62–0.73)***	0.65 (0.59–0.71)***	0.59 (0.46–0.77)***
White/Hispanic	0.87 (0.77–0.99)**	0.79 (0.69–0.90)***	1.21 (0.88–1.67)

Data are shown as hazard ratio (95% confidence interval).

DD, deceased donor; LD, living donor; PRA, panel reactive antibody; REF, reference.

** *P* < .01.

*** *P* < .001.

**Table 6:** Distribution of kidney transplant recipient race stratified by donor race for primary and repeat transplantation.

Recipient race	Initial transplant donor race, n (%)				
	Total	White, non-Hispanic	White, Hispanic	Black	Other
	N = 101 886	N = 69 476	N = 12 904	N = 17 300	N = 2206

White, non-Hispanic	51 984	43 894 (84.44%)	3389 (6.52%)	3734 (7.18%)	967 (1.86%)
Black	36 108	19 652 (54.43%)	3194 (8.85%)	12 430 (34.42%)	832 (2.30%)
White, Hispanic	13 794	5930 (42.99%)	6321 (45.82%)	1136 (8.24%)	407 (2.95%)

	Retransplant donor race, n (%)				
	Total	White, non-Hispanic	White, Hispanic	Black	Other
	N = 27 270	N = 19 403	N = 13 573	N = 3604	N = 660

White, non-Hispanic	16 043	13 473 (83.98%)	1227 (7.65%)	1002 (6.25%)	341 (2.13%)
Black	7489	4148 (55.39%)	838 (11.19%)	2313 (30.89%)	190 (2.54%)
White, Hispanic	3708	1782 (48.06%)	1508 (40.67%)	289 (7.79%)	129 (3.48%)

**Table 7:** Distribution of kidney transplant recipient race stratified by HLA mismatch for primary and repeat transplantation.

Recipient race	Initial transplant HLA mismatch, n (%)			
	Total	HLA mismatch = 0	HLA mismatch = 1–4	HLA mismatch = 5–6
	N = 95 318	N = 5959	N = 51 681	N = 37 678

White, non-Hispanic	47 841	3960 (8.28%)	28 289 (59.13%)	15 592 (32.59%)
Black	34 428	1016 (2.95%)	16 433 (47.73%)	16 979 (49.32%)
White, Hispanic	13 049	983 (7.53%)	6959 (53.33%)	5107 (39.14%)

	Retransplant HLA mismatch, n (%)			
	Total	HLA mismatch = 0	HLA mismatch = 1–4	HLA mismatch = 5–6
	N = 26 767	N = 3353	N = 14 890	N = 8524

White, non-Hispanic	15 769	2455 (15.57%)	8947 (56.74%)	4367 (27.69%)
Black	7371	413 (5.60%)	3879 (52.63%)	3079 (41.77%)
White, Hispanic	3627	485 (13.37%)	2064 (56.91%)	1078 (29.72%)

HLA, human leukocyte antigen.

**Table 8:** Multivariable Cox models for impact of various recipient sociodemographic and clinical factors on access to retransplant steps among patients who have previously received multiple kidney transplants (≥2), including rewaitlisting or retransplant.

Covariate	Access to waitlisting	Access from waitlist to DD or LD retransplant	Access from waitlist to DD retransplant^a^	Access from waitlist to LD retransplant^b^	Overall access to DD or LD retransplant^c^	Overall access to DD retransplant	Overall access to LD retransplant

Race/Ethnicity							
White, non-Hispanic	REF	REF	REF	REF	REF	REF	REF
Black	0.94 (0.91–0.97)***	0.70 (0.68–0.72)***	0.77 (0.75–0.80)***	0.46 (0.43–0.50)***	0.65 (0.63–0.67)***	0.74 (0.72–0.77)***	0.42 (0.39–0.45)***
White, Hispanic	0.94 (0.90–0.97)**	0.78 (0.75–0.81)***	0.78 (0.75–0.82)***	0.87 (0.80–0.95)***	0.72 (0.70–0.75)***	0.76 (0.73–0.80)***	0.70 (0.65–0.76)***
Sex							
Male	REF	REF	REF	REF	REF	REF	REF
Female	1.03 (1.00–1.05)*	1.01 (0.99–1.04)	0.98 (0.95–1.01)	1.07 (1.02–1.13)*	1.03 (1.01–1.06)*	1.01 (0.98–1.04)	1.10 (1.05–1.16)***
CAD	1.01 (0.94–1.08)	0.92 (0.86–0.99)*	0.96 (0.89–1.04)	0.85 (0.73–0.98)*	0.96 (0.90–1.03)	1.01 (0.93–1.09)	0.86 (0.75–0.98)*
CHF	0.89 (0.85–0.94)***	1.03 (0.98–1.08)	1.05 (0.99–1.11)	0.98 (0.88–1.09)	0.98 (0.93–1.03)	0.99 (0.93–1.04)	0.97 (0.88–1.07)
COPD	0.99 (0.88–1.11)	1.14 (1.01–1.28)*	1.27 (1.12–1.44)***	0.74 (0.55–1.00)*	1.06 (0.94–1.18)	1.18 (1.04–1.34)**	0.74 (0.56–0.96)*
Diabetes	0.90 (0.87–0.93)***	1.05 (1.02–1.09)**	1.11 (1.07–1.15)***	0.95 (0.89–1.01)	1.09 (1.06–1.13)***	1.10 (1.06–1.14)***	1.09 (1.03–1.16)**
PVD	0.94 (0.87–1.01)	0.97 (0.90–1.05)	0.99 (0.91–1.08)	0.88 (0.74–1.04)	0.97 (0.90–1.05)	0.97 (0.89–1.06)	0.96 (0.83–1.12)
Age at the time of first transplant	1.01 (1.01–1.01)***	1.00 (1.00–1.00)***	1.01 (1.01–1.01)***	0.99 (0.99–0.99)***	1.01 (1.01–1.01)***	1.01 (1.01–1.02)***	1.00 (1.00–1.00)***
BMI	1.00 (1.00–1.00)	1.00 (1.00–1.00)	1.00 (1.00–1.00)	1.00 (1.00–1.00)	1.00 (1.00–1.00)	1.00 (1.00–1.00)	1.00 (1.00–1.00)
Years from ESRD initiation to first transplant	0.99 (0.99–1.00)*	0.99 (0.98–0.99)***	1.02 (1.01–1.02)***	0.84 (0.82–0.86)***	0.97 (0.96–0.97)***	1.01 (1.00–1.01)	0.80 (0.78–0.81)***
Years from first transplant to graft failure	1.03 (1.02–1.03)***	1.02 (1.01–1.02)***	1.00 (1.00–1.01)	1.04 (1.04–1.05)***	1.03 (1.02–1.03)***	1.01 (1.01–1.02)***	1.04 (1.04–1.05)***
Percentage below poverty	1.00 (0.99–1.00)***	1.00 (1.00–1.00)	1.00 (1.00–1.00)*	0.99 (0.99–0.99)***	1.00 (1.00–1.00)**	1.00 (1.00–1.00)	0.99 (0.98–0.99)***
Insurance							
Group health insurance	1.07 (1.04–1.10)***	1.05 (1.02–1.09)**	0.97 (0.93–1.00)	1.24 (1.17–1.32)***	1.10 (1.07–1.14)***	1.01 (0.98–1.05)	1.29 (1.22–1.36)***
Medicaid	0.95 (0.92–0.99)**	0.97 (0.93–1.00)	1.02 (0.98–1.07)	0.78 (0.71–0.85)***	0.95 (0.91–0.98)**	1.01 (0.97–1.06)	0.75 (0.69–0.81)***
Medicare or other insurance	REF	REF	REF	REF	REF	REF	REF
No health insurance	0.85 (0.70–1.04)	1.12 (0.91–1.38)	1.25 (1.00–1.56)*	0.67 (0.36–1.25)	1.00 (0.82–1.24)	1.12 (0.89–1.39)	0.61 (0.34–1.07)
Unknown	0.81 (0.75–0.87)***	0.97 (0.90–1.04)	1.02 (0.94–1.10)	0.76 (0.63–0.92)**	0.84 (0.78–0.90)***	0.87 (0.81–0.94)***	0.68 (0.58–0.80)***
Recipient blood type							
A	REF	REF	REF	REF	REF	REF	REF
O	1.00 (0.97–1.03)	0.86 (0.83–0.88)***	0.83 (0.80–0.86)***	0.93 (0.88–0.98)*	0.87 (0.85–0.90)***	0.85 (0.82–0.88)***	0.93 (0.88–0.98)**
B	1.06 (1.02–1.11)**	0.82 (0.79–0.86)***	0.74 (0.70–0.78)***	1.12 (1.03–1.22)**	0.87 (0.84–0.91)***	0.79 (0.75–0.83)***	1.13 (1.04–1.22)**
AB	1.02 (0.96–1.09)	1.17 (1.10–1.25)***	1.26 (1.18–1.35)***	0.95 (0.83–1.08)	1.11 (1.05–1.18)***	1.20 (1.12–1.29)***	0.93 (0.82–1.04)
PRA							
PRA = 0	—	REF	REF	REF	—	—	—
0 < PRA ≤80	—	0.72 (0.70–0.74)***	0.95 (0.91–0.99)**	0.43 (0.40–0.45)***	—	—	—
PRA >80	—	0.56 (0.54–0.59)***	0.72 (0.68–0.76)***	0.30 (0.27–0.34)***	—	—	—
Status 7	—	0.60 (0.58–0.62)***	0.38 (0.36–0.40)***	1.44 (1.36–1.53)***	—	—	—
Years from graft failure to waitlisting	—	1.07 (1.06–1.08)***	1.10 (1.10–1.11)***	0.89 (0.87–0.92)***	—	—	—

Data are shown as hazard ratio (95% confidence interval).

BMI, body mass index; CAD, coronary artery disease; CHF, congestive heart failure; COPD, chronic obstructive pulmonary disease; DD, deceased donor; ESRD, end-stage renal disease; LD, living donor; PRA, panel reactive antibody; PVD, peripheral vascular disease; REF, reference.

* *P* < .05.

** *P* < .01.

*** *P* < .001.

## Data Availability

The data that support the findings of this study are available on request from the corresponding author.

## Appendix A. Supplementary data

Supplementary data to this article can be found online at https://doi.org/10.1016/j.ajt.2026.03.010.

## Abbreviations:

CI: confidence interval
cPRA: calculated panel reactive antibody
ESRD: end-stage renal disease
HLA: human leukocyte antigen
HR: hazard ratio
IQR: interquartile range
PRA: panel reactive antibody
SAF: Standard Analysis File
USRDS: United States Renal Data System
