# Supplementary material for: Racial and immunologic disparities impact access to kidney rewaitlisting and retransplantation
Source: Am J Transplant. Author manuscript; Available in PMC 2026 Sep 28. (PMC13617515; doi:10.1016/j.ajt.2026.03.010)
Supplement: Supplementary file 1 [file NIHMS2203302-supplement-1.docx]

Supplemental Table 1: Characteristics of preemptively re-waitlisted patients with a failed kidney allograft, 1995-2022

| **Characteristic** | **Overall  N=13,561** | **White, non-Hispanic N=8187 (60.37)** | **Black N=3871 (28.55)** | **White, Hispanic N=1503 (11.08)** | **P-value** |
| --- | --- | --- | --- | --- | --- |
| Cause of ESRD |  |  |  |  |  |
| Diabetes | 2,334 (17.21) | 1,484 (18.13) | 608 (15.71) | 242 (16.1) | 0.002 |
| Hypertension | 2,088 (15.40) | 669 (8.17) | 1,197 (30.92) | 222 (14.77) | <.001 |
| Glomerulonephritis | 4,492 (33.12) | 2,613 (31.92) | 1,300 (33.58) | 579 (38.52) | <.001 |
| Cystic kidney | 1278 (9.42) | 1,057 (12.91) | 128 (3.31) | 93 (6.19) | <.001 |
| Other urologic | 742 (5.47) | 570 (6.96) | 96 (2.48) | 76 (5.06) | <.001 |
| Other cause | 1,875 (13.83) | 1,342 (16.39) | 354 (9.14) | 179 (11.91) | <.001 |
| Unknown cause | 710 (5.24) | 426 (5.2) | 176 (4.55) | 108 (7.19) | <.001 |
| Missing cause | 42 (0.31) | 26 (0.32) | 12 (0.31) | 4 (0.27) | 0.947 |
| Insurance |  |  |  |  |  |
| Group health | 6,048 (44.60) | 4,134 (50.49) | 1,419 (36.66) | 495 (32.93) | <.001 |
| Medicaid | 2,539 (18.72) | 1,060 (12.95) | 947 (24.46) | 532 (35.4) | <.001 |
| Medicare/Other | 4,415 (32.56) | 2,753 (33.63) | 1,271 (32.83) | 391 (26.01) | <.001 |
| No health insurance | 210 (1.55) | 76 (0.93) | 104 (2.69) | 30 (2) | <.001 |
| Unknown | 349 (2.57) | 164 (2) | 130 (3.36) | 55 (3.66) | <.001 |
| Sex |  |  |  |  |  |
| Male | 7,747 (57.13) | 4,695 (57.35) | 2,220 (57.35) | 832 (55.36) | 0.339 |
| Female | 5,814 (42.87) | 3,492 (42.65) | 1,651 (42.65) | 671 (44.64) |  |
| CAD, N (%) | 707 (5.55) | 497 (6.44) | 147 (4.09) | 63 (4.39) | <.001 |
| CHF, N (%) | 1,098 (8.62) | 624 (8.09) | 371 (10.32) | 103 (7.18) | <.001 |
| PVD, N (%) | 522 (4.10) | 349 (4.52) | 123 (3.42) | 50 (3.49) | 0.011 |
| COPD, N (%) | 184 (1.44) | 126 (1.63) | 44 (1.22) | 14 (0.98) | 0.069 |
| Diabetes, N (%) | 3,669 (27.59) | 2,135 (26.5) | 1,118 (29.72) | 416 (28.18) | 0.001 |
| Age at first transplant, Mean (SD) | 38.31 (15.28) | 38.3 (15.7) | 39.89 (13.93) | 34.28 (15.53) | <.001 |
| BMI, Mean (SD) | 27.15 (20.99) | 27.04 (25.51) | 27.71 (11.9) | 26.33 (7.37) | 0.078 |
| Years from 1st transplant to graft failure, Mean (SD) | 1.87 (2.39) | 1.28 (1.92) | 3 (2.78) | 2.2 (2.49) | <.001 |
| Years from failure to retransplant, Mean (SD) | 2.54 (2.67) | 2.23 (2.51) | 3.37 (2.95) | 2.74 (2.64) | <.001 |
