# Supplementary material for: Racial and immunologic disparities impact access to kidney rewaitlisting and retransplantation
Source: Am J Transplant. Author manuscript; Available in PMC 2026 Sep 28. (PMC13617515; doi:10.1016/j.ajt.2026.03.010)
Supplement: Supplementary file 2 [file NIHMS2203302-supplement-2.docx]

Supplemental Table 2 – Multivariable Cox models for impact of various recipient sociodemographic and clinical factors on access to retransplant steps, including re-waitlisting or re-transplant

|  | **FAILED PRIMARY KIDNEY TRANSPLANT RETURNED TO DIALYSIS** | **FAILED PRIMARY KIDNEY TRANSPLANT WITH PREEMPTIVE RETRANSPLANT** | |
| --- | --- | --- | --- |
| **Covariate** | **Access from Waitlist to DD or LD Retransplant** | **Access from Waitlist to DD or LD Retransplant** |  |
| Race/Ethnicity |  |  |  |
| White, non-Hispanic | REF | REF |  |
| Black | 0.63 (0.60-0.66)*** | 0.68 (0.64-0.72)*** |  |
| White, Hispanic | 0.76 (0.73-0.80)*** | 0.75 (0.69-0.81)*** |  |
| Sex |  |  |  |
| Male | REF | REF |  |
| Female | 1.02 (0.99-1.05) | 1.03 (0.99-1.08) |  |
| CAD | 1.03 (0.94-1.12) | 0.78 (0.69-0.88)*** |  |
| CHF | 1.01 (0.95-1.07) | 0.85 (0.77-0.94)** |  |
| COPD | 1.13 (0.98-1.29) | 0.85 (0.67-1.07) |  |
| Diabetes | 1.00 (0.96-1.04) | 0.79 (0.75-0.84)*** |  |
| PVD | 0.96 (0.87-1.05) | 0.84 (0.73-0.97)* |  |
| Age at the time of first transplant | 0.99 (0.99-0.99)*** | 0.99 (0.99-0.99)*** |  |
| BMI | 1.00 (1.00-1.00) | 1.00 (1.00-1.00) |  |
| Years from ESRD initiation to 1st transplant | 0.97 (0.96-0.97)*** | 0.92 (0.91-0.94)*** |  |
| Years from 1st transplant to graft failure | 1.00 (0.99-1.00) | 1.02 (1.01-1.02)*** |  |
| Percent below poverty | 1.00 (0.99-1.00)*** | 1.00 (0.99-1.00) |  |
| Insurance |  |  |  |
| Medicare or other insurance | REF | REF |  |
| Group health insurance | 1.03 (0.99-1.07) | 1.25 (1.18-1.32)*** |  |
| Medicaid | 0.92 (0.88-0.96)*** | 0.87 (0.81-0.94)*** |  |
| No health insurance | 0.89 (0.70-1.13) | 1.09 (0.69-1.72) |  |
| Unknown | 0.90 (0.83-0.98)* | 1.20 (1.00-1.45) |  |
| Recipient blood type |  |  |  |
| A | REF | REF |  |
| O | 0.83 (0.80-0.86)*** | 0.81 (0.77-0.85)*** |  |
| B | 0.70 (0.66-0.74)*** | 0.76 (0.70-0.82)*** |  |
| AB | 1.15 (1.07-1.25)*** | 1.21 (1.08-1.35)*** |  |
| PRA |  |  |  |
| PRA=0 | REF | REF |  |
| 0<PRA<=80 | 0.67 (0.65-0.70)*** | 0.79 (0.75-0.83)*** |  |
| PRA>80 | 0.42 (0.40-0.45)*** | 0.64 (0.58-0.71)*** |  |
| Status 7 | 0.13 (0.13-0.14)*** | 0.25 (0.24-0.27)*** |  |
| Years from graft failure to waitlisting | 1.01 (1.00-1.02) | -- |  |

ESRD: end stage renal disease; CAD: coronary artery disease; CHF: congestive heart failure; PVD: peripheral vascular disease; COPD: chronic obstructive pulmonary disease; PRA: panel reactive antibody

(*p-value<0.05 **p-value<0.01 ***p-value<0.001)
