# Supplementary material for: Racial and immunologic disparities impact access to kidney rewaitlisting and retransplantation
Source: Am J Transplant. Author manuscript; Available in PMC 2026 Sep 28. (PMC13617515; doi:10.1016/j.ajt.2026.03.010)
Supplement: Supplementary file 3 [file NIHMS2203302-supplement-3.docx]

Supplemental Text:

We developed an a priori Causal Direct Acyclic Graph (CDAG) model based on known factors associated with access to transplantation, both initial and subsequent retransplantation, as shown in Supplemental Figure 1. Our analysis uses a sequential adjustment strategy to distinguish between different causal effects. First, confounders (Backdoor Paths), are variables like Age, Sex, and Blood Group. They are common causes (or associated with the cause) of both the exposure (Race) and the outcome (Retransplant Access). Adjusting for these is necessary to satisfy the "backdoor criterion" and obtain an unbiased estimate of the total association. Second, mediators (Mechanisms), including variables like SES and immunologic sensitization (PRA). They are consequences of race or systemic factors that subsequently lead to the outcome. We adjust for these in our "fully adjusted" models to estimate the Direct Effect—answering whether a racial disparity persists even after accounting for socioeconomic and immunologic mechanisms. The covariates in our models are grouped into four specific pathways. (1) The Demographic Pathway (Confounders): Age and Sex: Essential for baseline risk adjustment. Blood Group: Inherited differences in ABO frequencies affect ability for transplant matching. Controlling for this ensures the "race effect" is not a byproduct of biological blood type distribution. (2) The Socioeconomic Pathway (Mediators): Insurance and Neighborhood Poverty: These represent the structural/social mechanisms of systemic racism. Our results show these factors primarily explain the gap in re-waitlisting. (3) The Immunologic Pathway (Mediators): Initial HLA Mismatch -> Peak PRA: Because the donor pool is ~70% White, minority recipients often receive more mismatched organs in their first transplant. This leads to higher sensitization (PRA), which acts as a major biological barrier to the second transplant. (4) The Clinical Pathway: Comorbidities (Diabetes, CAD, CHF, etc.): Included to ensure that observed disparities exist independent of clinical candidate fitness or surgical risk.
